# Artificial intelligence vs. emergency physicians: who diagnoses better?

**DOI:** 10.1590/1806-9282.20250546

**Published:** 2025-12-05

**Authors:** Ali İhsan Kilci, Ramazan Azim Okyay, Erhan Kaya, Muhammed Semih Gedik, Hakan Hakkoymaz, Murat Tepe

**Affiliations:** 1 Kahramanmaraş Sütçü İmam University, Emergency Medicine – Kahramanmaraş, Turkey.; 2 Kahramanmaraş Sütçü İmam University, Department of Public Health – Kahramanmaraş, Turkey.

**Keywords:** Emergency medicine, Artificial intelligence, Diagnosis

## Abstract

**OBJECTIVE:**

The aim of this study was to compare the diagnostic accuracy and initial diagnostic test selection capabilities of large language models with an experienced emergency medicine specialist in simulated emergency department scenarios.

**METHODS:**

A series of brief case presentations were created by an expert committee to reflect real-world emergency conditions. Each brief case presentation included clinical history and physical examination findings but excluded laboratory and imaging data. The study compared the diagnostic accuracy and initial test selection performance of an emergency medicine specialist with three different large language model versions: ChatGPT-4, ChatGPT-4o, and ChatGPT-3.5-mini. The accuracy of responses was assessed based on predefined correct diagnoses and appropriate first-line tests. Statistical comparisons were conducted using the Cochran-Q test and McNemar test.

**RESULTS:**

The diagnostic accuracy rates were 92% for the human expert, 97% for ChatGPT-4, and 99% for both ChatGPT-4o and ChatGPT-3.5-mini (p=0.039 for ChatGPT-4o and ChatGPT-3.5-mini vs. human expert). The accuracy of initial diagnostic test selection was 88% for the human expert, 80% for ChatGPT-4, 87% for ChatGPT-4o, and 89% for ChatGPT-3.5-mini (p>0.05 for all comparisons). The most frequent diagnostic errors were related to cardiovascular (7/13) and gastrointestinal (4/13) cases.

**CONCLUSIONS:**

Large language models demonstrated acceptable diagnostic accuracy, outperforming the human expert in diagnosis while performing comparably in selecting initial diagnostic tests. These findings suggest that artificial intelligence models could serve as valuable decision-support tools in emergency medicine. However, further research is needed to evaluate their performance in real-world clinical settings.

## INTRODUCTION

Emergency medicine necessitates rapid decision-making with limited initial data, where diagnostic accuracy directly affects patient outcomes and resource use. Emergency physicians must balance time constraints, cognitive workload, and the need for precise diagnoses, contributing to significant stress and diagnostic errors affecting millions annually^1,2^.

Artificial intelligence (AI), particularly large language models (LLMs), has shown potential in healthcare applications, including clinical reasoning and decision support^3,4^. While AI has been evaluated for tasks such as image interpretation and hospital admission predictions, its role in emergency diagnostic reasoning remains underexplored^5,6^.

Clinical diagnosis involves complex cognitive processes honed through years of experience. The extent to which LLMs can replicate or enhance expert reasoning is a critical question^7^. Traditional clinical decision support systems have shown variable efficacy, often due to poor workflow integration. However, modern LLMs, capable of processing medical narratives and providing explanatory reasoning, may offer improved decision support^8,9^.

This study compares the diagnostic performance and initial test selection of multiple LLM versions with an experienced emergency physician using standardized case presentations. By simulating real-time clinical encounters, it evaluates AI's role in emergency diagnostic reasoning, addressing key questions about its capabilities and limitations in acute care settings.

## METHODS

### Study design and objective

This cross-sectional study aimed to compare the diagnostic performance and initial diagnostic test selection capabilities of LLMs versus an emergency medicine specialist (human expert [HE]). Case scenarios of comparable complexity to real clinical cases were developed, and the accuracy of participants’ diagnostic decision-making processes was measured.

### Data source

The study utilized brief case presentations (BCPs) created by an expert committee (EC) consisting of two associate professors and one assistant professor of emergency medicine (HH, MSG, and MT). These BCPs included only clinical presentations and physical examination findings and were developed between December 1, 2024, and February 15, 2025.

The BCPs were designed to represent conditions that are either common or critically important in emergency departments and include clear diagnostic criteria. According to the Healthcare Cost and Utilization Project (HCUP) Statistical Brief 286, emergency department admissions at the national level comprise 18.0% circulatory system disorders, 13.3% digestive system disorders, and 11.9% respiratory system disorders. Following this distribution, our study primarily included case scenarios related to cardiovascular and gastrointestinal systems^10^. When the case scenarios were classified according to the Emergency Severity Index (ESI), it was seen that there were 3, 41.4, 38.4, 16.2, and 1% cases in ESI 1-2-3-4-5 classes, respectively.

Cerebrovascular disease was the most frequently included diagnosis (five cases), followed by acute appendicitis (four cases). Other conditions such as acute pancreatitis, acute peripheral arterial occlusion, aortic dissection, acute hepatitis, cardiac tamponade, mesenteric ischemia, pneumothorax, and pulmonary embolism were each included as three cases (Table 1).

**Table 1:** Diagnostic accuracy.

Participants	Responses		p^a^
	Correct (%)	Incorrect (%)	
Human expert	92	8	0.014
ChatGPT-4	97	3	
ChatGPT-4o	99	1	
ChatGPT o3-mini	99	1	

a Cochran-Q test; statistical difference was found between paired measurements.

### Participants and comparison groups

#### Human expert

An emergency medicine specialist (assistant professor) with at least 5 years of experience participated in the study.

#### Artificial Intelligence Models (LLMs)

- ChatGPT-4: The older model available during the study period (Mar 2023).
- ChatGPT-4o: A newer version of ChatGPT-4 with different parameter settings or slight modifications, supporting image and voice commands (May 2024).
- ChatGPT o3-mini: The latest version available during the study period (Jan 2025).

All models were accessed via the OpenAI platform during the study period. No custom fine-tuning or modifications were applied.

#### Implementation procedure

Each BCP was presented in a standard format and in Turkish language, including the patient's age, gender, chief complaint, onset duration, and any additional medical history. Physical examination findings included vital signs (blood pressure, pulse, respiratory rate, and temperature) and significant positive/negative examination results. Laboratory or imaging findings were intentionally omitted to evaluate only the initial diagnostic test selection based on clinical and physical examination data.

Both the HE and LLM models were asked to provide the most likely diagnosis (considering emergency conditions) and the first diagnostic test required to reach this diagnosis. Participants were reminded that timing is critical in establishing the "most likely diagnosis" and that they should consider emergency medicine prioritization.

The prompt ordered for LLMs to evaluate the cases was as follows: "For a study evaluating diagnostic capabilities of LLMs in emergency medicine, you will be provided with simulated patient case scenarios. Please approach each case as if you are an emergency medicine physician. For each scenario; first specify the most probable diagnosis, secondly identify the single most appropriate initial diagnostic test to confirm or exclude this diagnosis." However, since the relevant case scenarios were in Turkish language, this prompt was also translated into Turkish and presented to HEs and LLMs for evaluation. The HE evaluated the BCPs one by one and provided written responses for each. Responses from LLMs were obtained using the same prompt text and providing the same input information. The accuracy and appropriateness of the responses were evaluated by the EC.

#### Accuracy criteria

For the BCPs, exact matches or clinically acceptable approximations to the predetermined diagnosis by the EC were considered "correct." The correct prioritization of the fundamental test required to reach the mentioned diagnosis under emergency conditions was expected. For example, while "abdominal ultrasound" was often expected as the first test for acute abdominal pain, suggesting advanced procedures such as "abdominal MRI" was considered incorrect.

In cases requiring direct intervention instead of diagnostic testing, both the first test response and the emergency intervention recommendation were accepted as correct. For example, in hypoglycemia, both blood glucose measurement and the recommendation to administer glucose to the patient were considered correct.

### Statistical analysis

The rate of correct diagnosis and correct initial diagnostic test selection was calculated for each participant group (HE, ChatGPT-4, ChatGPT-4o, and ChatGPT o3mini). Differences between paired measurements were analyzed using the Cochran-Q test. The McNemar test was used for paired posthoc analyses of consecutive measurements. The accuracy rates of the raters are given with 95%CI. A p<0.05 was considered statistically significant for primary analysis. However, the Bonferroni correction was applied for the p-value in posthoc analyses, and p<0.008 was considered significant.

### Ethical approval

Ethics committee approval was not obtained for this study as it was conducted with case presentation scenarios created by the EC and did not involve any animals, human participants, or personal information.

## RESULTS

The diagnostic accuracy rates were calculated for each participant group (HE and three different LLM models). HE, ChatGPT-4, ChatGPT-4o, and ChatGPT o3-mini responded to cases with 92.0% (95%CI 84.8–96.5%), 97.0% (95%CI 91.5–99.4%), 99.0% (95%CI 94.6–99.98%), and 99.0% (95%CI 94.6–99.98%) accuracy, respectively. There was a difference in diagnostic accuracy between consecutive measurements of the four raters (p=0.014; Cochran-Q test for multiple consecutive measurements). In the posthoc comparison between ChatGPT-4o and ChatGPT o3-mini with HE, the McNemar test result was found to be p=0.039 for both comparisons; however, for the subgroup analysis, the Bonferroni correction was applied and the p<0.008 level was taken into account, and the difference was not considered statistically significant (Table 1).

The accuracy rates of participants in selecting the first diagnostic test were calculated. ChatGPT o3-mini responded with 89% (95%CI 81.2–94.4%) accuracy, HE with 88% (95%CI 80.0–93.6%) accuracy, and ChatGPT-4o with 87% (95%CI 78.8–92.9%) accuracy. Although ChatGPT-4 model's accuracy rate of 80% (95%CI 70.8–87.3%) was lower compared to the HE, this difference was also not statistically significant (p=0.208; Cochran-Q test) (Table 2).

**Table 2:** First diagnostic test accuracy.

Participants	Responses		p^a^
	Correct (%)	Incorrect (%)	
Human expert	88	12	0.208
ChatGPT-4	80	20	
ChatGPT-4o	87	13	
ChatGPT o3-mini	89	11	

a Cochran-Q test; no statistical difference was found between paired measurements.

When examining the incorrect answers given by participants, it is noteworthy that the most incorrect answers were related to the cardiovascular system (7/13). The second most incorrect answers were related to the gastrointestinal and hepatic system (4/13) (Table 3).

**Table 3:** Distribution of misdiagnoses.

Participants	Responses	
	Correct diagnosis	Participants’ incorrect diagnosis
Human expert	Penil fracture Acute limb ischemia Rectus sheath hematoma Stevens-Johnson syndrome Mesenteric ischemia Addison's disease Cardiac tamponade Acute liver failure	Lumbal fracture Chronic arterial occlusion Abdominal aorta rupture Pemphigus vulgaris Ileus Inflammatory bowel disease Vena cava superior syndrome Hepatic encephalopathy
ChatGPT4	Pulmonary embolism Pulmonary edema phlegmasia cerulea dolens	Postpartum cardiomyopathy Acute heart failure Acute limb ischemia
ChatGPT-4o	Pulmonary edema	Acute heart failure
ChatGPT o3-mini	Acute hepatitis	Choledocholithiasis

## DISCUSSION

Based on the findings of our study, which examined the diagnostic accuracy and initial diagnostic test performance of AI language models and expert physicians in emergency department cases weighted and categorized by primary diagnostic groups, HEs achieved a diagnostic accuracy of 92%, while ChatGPT-4 reached 97%, and both ChatGPT-4o and ChatGPT-3.5-mini achieved 99% accuracy. Ultimately, there is no clear evidence to suggest that LLM is more successful in terms of diagnosic capabilities than HE.

In the increasingly digitized world, natural language AI has emerged as a transformative technology that enables computers to understand and respond to human language. This advancement has the potential to enhance medical practice by providing intuitive and natural user experiences and responses. Such AI-driven capabilities could find new applications in emergency medicine to improve the efficiency and accuracy of patient care^11^. In emergency departments, diagnostic decision tools play a critical role in screening and classifying patients, making them essential for initial clinical interventions. Research has been conducted on developing potential new diagnostic decision support applications and data mining techniques. Machine learning models have demonstrated high accuracy in the early prediction and diagnosis of high-risk conditions such as acute kidney injury (AKI), sepsis, pneumonia, and influenza, allowing for timely interventions to prevent complications associated with disease progression in emergency settings. According to previous studies, logistic regression models have exhibited high diagnostic accuracy, ranging from 70 to 90%, with reported accuracy rates of 99.1% for AKI and 89.1% for chronic obstructive pulmonary disease and asthma. A study conducted in an emergency department on a large cohort of syncope patients demonstrated that an AI algorithm achieved 92.2% sensitivity and a positive predictive value of 47.4% in automatically identifying cases^12–14^.

Our findings show an acceptable level of diagnostic accuracy when evaluated in line with the existing literature, although LLM models are not clearly superior to HE. This study provided an opportunity to compare the diagnostic capabilities of AI applications with those of expert physicians. Our results showed that AI models exhibited similar diagnostic accuracy than HEs. The results of our study should be interpreted together with the fact that the analyzed cases were obtained from the literature. Given that AI models can scan and analyze vast amounts of online medical data, they may outperform physicians in interpreting clinical presentations.

While LLMs demonstrated high accuracy, their performance may vary in real-world clinical practice where incomplete data, time constraints, and emotional factors affect decision-making. Furthermore, unlike human clinicians, AI models cannot justify their decisions in legal or ethical contexts, raising concerns regarding accountability. Despite the promising performance of AI models, our results underscore the importance of human expertise in clinical decision-making, particularly in implementing evidence-based practices that guide medical interventions.

On the other hand, when examining the accuracy of initial diagnostic tests, HEs demonstrated an accuracy rate of 88%, while ChatGPT-4 achieved 80%, ChatGPT-4o reached 87%, and ChatGPT-3.5-mini attained 89%. These findings suggest that although not statistically significant, LLMs may have lower accuracy in initial diagnostic assessments compared to HEs. Wrong selection of the first diagnostic test may lead to delayed or incorrect diagnoses and may cause harm to the patient these results should be considered carefully. In the field of emergency medicine, research on AI modeling has predominantly focused on diagnosis and triage. Diagnostic studies have primarily centered on prediction and decision support, whereas triage-specific research has encompassed applications such as mortality prediction, patient outcomes, hospitalization forecasting, severity assessment, and emergency care prognosis^15^. A literature review covering the period from 2015 to 2021 highlighted the increasing number of publications on AI in emergency care, emphasizing AI's potential role in decision-making, workflow optimization, and operational management. However, concerns regarding inappropriate algorithm selection, data privacy, and security underscore the necessity for improved research standards in AI implementation and reporting. Consequently, these concerns contribute to ongoing reservations about the widespread adoption of AI in clinical practice^16–18^. A study on AI-based triage modeling for patients presenting with acute abdominal pain demonstrated that the AI system could accurately and independently triage patients at levels 3 and 4, achieving an acceptable level of accuracy^19^. In a study conducted by Kang et al., which utilized development data from the Korean National Emergency Department Information System, an AI model was designed to predict critical care needs in adult emergency patients. The model incorporated variables such as age, sex, chief complaint, duration from symptom onset to arrival, trauma history, and initial vital signs. Results indicated that the AI model outperformed traditional triage tools, achieving an area under the curve (AUC) of 0.867^20^.

In the study by Kanjee et al., it was found that a generative AI model provided the correct diagnosis in differential diagnosis in 64% of difficult cases^21^. Using the New England Journal of Medicine clinicopathologic case conferences, Fritz et al. found that with two such models, the correct diagnosis was determined in 58–68% of cases^22^. The acceptability of these rates is a matter of debate, and it is clear that studies are needed on this subject. Additionally, in today's conditions, it does not seem possible to completely eliminate the concept of hallucination while maintaining high performance standards in LLMs^23^.

### Limitations

First, the study was conducted using only simulated case scenarios and did not include actual patient interactions and emergency room dynamics. Therefore, the real-world generalizability of the results may be limited. While this approach may present some practical limitations, it provides a standardized methodology that offers valuable insights. Second, a single emergency medicine physician was used as the HE. Extended studies involving multiple physicians with varying levels of experience are needed.

Since the learning processes of the LLMs are not open source, they have not been possible for researchers to control these processes and it cannot be assessed whether the models used were indirectly trained on the study scenarios, posing a potential risk of data leakage. Third, the use of proprietary AI models limits transparency about training data and architecture, which may influence diagnostic outcomes. Fourth, future studies should compare LLMs with traditional clinical decision support systems such as Isabel or Watson, which are already integrated into some emergency department workflows. Such comparisons would offer clearer insights into the added value or limitations of generative AI.

Another limitation is that treatment decisions and long-term patient outcomes could not be evaluated in the study, since the cases were not real-time cases. Although no real patient data was used, diagnostic studies using AI should be evaluated according to the World Health Organization (WHO) ethical principles.

## CONCLUSION

This study demonstrates that LLMs exhibit similar diagnostic accuracy, with the potential to surpass HEs in certain scenarios. AI models performed particularly acceptable in overall diagnostic accuracy, while HEs provided more reliable results in initial diagnostic testing. These findings reinforce the indispensable role of HEs in clinical assessments and practical diagnostic interventions, while also highlighting AI's potential as an assistive tool in critical emergency diagnosis and initial diagnostic evaluations. Further research is necessary to validate these findings.

## Data Availability

The datasets generated and/or analyzed during the current study are available from the corresponding author upon reasonable request.
